# Clinical features and disease severity in patients with mosaic neurofibromatosis type 1: a single-center study and literature review

**DOI:** 10.1186/s13023-021-01796-3

**Published:** 2021-04-14

**Authors:** C. Ejerskov, M. Raundahl, P. A. Gregersen, M. M. Handrup

**Affiliations:** 1 Centre for Rare Diseases, Department of Paediatrics and Adolescent Medicine, Aarhus University Hospital, Palle Juul-Jensens Boulevard 99, 8200 Aarhus N, Denmark; 2 Department of Clinical Genetics and Centre for Rare Diseases, Department of Paediatrics and Adolescent Medicine, Aarhus University Hospital, Brendstrupgaardsvej 21 C, 8200 Aarhus N, Denmark

**Keywords:** Neurofibromatosis type 1, Neurofibroma, Plexiform neurofibroma, Mosaicism, Mosaic, *NF1* gene, NF1 guideline

## Abstract

**Background:**

The mosaic form of neurofibromatosis type 1 (NF1) is called mosaic NF1 (MNF1). No specific MNF1 follow-up guidelines exist. It is debatable if patients with MNF1 should be clinically examined and undergo follow-up in accordance with the standard NF1 guidelines, as MNF1 patients more often may develop more benign phenotypes and thereby less disease-associated complications including cognitive impairment. We discussed the need for a specific MNF1 follow-up guideline with focus on frequency of plexiform neurofibromas and NF1-associated complications.

**Method:**

A systematic retrospective data collection in a MNF1 cohort from one of two Danish national centers of NF1 Expertise was completed. Data collected included demographics, clinical features including NF1 diagnostic criteria and NF1-associated complications. Recent literature in the field was reviewed.

**Results:**

We identified 17 patients with MNF1 with a median age of 37 years [4; 66]. Eleven (65%) were females. Five patients (30%) had a plexiform neurofibroma. The median age at detection of plexiform neurofibroma was 30 years [14; 60]. Nine (53%) had at least one NF1-related complication; scoliosis, hypertension, ADHD, learning disability, language delay, autism and delay in gross and fine motor function development. We reviewed nine articles. In total, 126 cases were described within three case-series. Nineteen (15%) had a plexiform neurofibroma and in total, 23 NF1-associated complications were reported including language delay, learning disability and skeletal abnormalities. Furthermore, from the literature it was evident that the diagnosing of MNF1 varies among physicians and across countries.

**Conclusion:**

Patients with MNF1 present with plexiform neurofibromas and other NF1-related complications with a frequency requiring that follow-up of MNF1 patients should be in accordance with the standard NF1 guideline in both childhood and adulthood. Physicians should be aware of cognitive impairment as a complication to MNF1. To develop a specific MNF1 follow-up guideline, there is a need for an international consensus on the diagnostic criteria for MNF1 and a follow-up study conducted in a larger MNF1 cohort.

## Introduction

Neurofibromatosis type 1 (NF1) is a complex neurocutaneous disorder caused by loss of function variants and microdeletions in the *NF1* gene coding for the protein *neurofibromin* [1, 2]. With an incidence between 1:2000 and 1:3000, NF1 is one of the more common rare diseases [3, 4]. Fifty percent of NF1 cases are hereditary and the remaining are caused by de novo* NF1* variants [5]. NF1 is characterized by the clinical features café-au-lait (CAL) spots, axillary and inguinal freckling, neurofibromas and Lisch nodules; these features are present in the vast majority of patients. Other clinical features in a significant proportion of patients include plexiform neurofibromas and different NF1-associated complications including learning disabilities, skeletal dysplasia, and an increased risk of specific malignancies [6].

NF1 exists as a mosaic form called mosaic NF1 (MNF1) caused by postzygotic pathogenic variants in *NF1*. *NF1* spans approximately 350 Kb of genomic DNA and has one of the highest mutation rates, resulting in a relatively high frequency of mosaicism [7, 8]. Disease-associated manifestations of MNF1 are limited to the affected area of the body, from a small part to half the body. Manifestations are usually unilateral but may appear bilaterally, either in a symmetric or asymmetrical form [8].

It is debatable if patients with MNF1 should be clinically examined and undergo follow-up in accordance with the standard NF1 guidelines, as patients with MNF1 may more often develop more benign phenotypes and have a much lower risk of developing plexiform neurofibromas and NF1-associated complications such as cognitive impairment and malignancy [5, 8–10]. A systematic review of 157 cases of MNF1 published between 1977 and 2012 found that 11.5% had plexiform neurofibromas, 29% had NF1-associated complications and 13% had a risk of malignancy, especially if they had neurofibromas [11]. Between 30 and 50% of patients with NF1 develop plexiform neurofibromas [6, 12]. Plexiform neurofibromas are congenital benign peripheral nerve sheath tumors localized in the head, neck, limbs, truncus, thorax as well as the abdominal and pelvic cavity. It is estimated that large lesions account for 5% of all plexiform neurofibromas [12]. Currently, the only standard treatment for plexiform neurofibromas is surgical resection. However, given the infiltrative nature and size of plexiform neurofibromas, complete surgical removal is usually not possible [13]. Last year, selumetinib, a small molecule MEK inhibitor which has been proven to decrease tumor volume of plexiform neurofibromas in children with NF1 was approved by US Food and Drug Administration to treat congenital and inoperable plexiform neurofibromas in children with NF1 [14]. Plexiform neurofibromas can transform into malignant peripheral nerve sheet tumors (MPNST), which is one of the most common NF1-related malignancies [15]. MPNST is an aggressive sarcoma, difficult to detect and with a poor prognosis. It is rare in the general population (incidence 0.001%), but the incidence in patients with NF1 has been found to be 2–5% with a cumulative lifetime risk of 8–13% [16].

While there are internationally accepted guidelines for the diagnosis and management of NF1, there are no specific guidelines on MNF1 [6, 8]. Due to the new treatment of plexiform neurofibromas, selumetinib, it is of interest to report plexiform neurofibromas in patients with MNF1. We present data from a cohort of patients with MNF1 with focus on the proportion of neurofibromas, plexiform neurofibromas, MPNST, and other NF1-associated complications. We also present a review of literature on MNF1 published between 2013 and 2020 with focus on plexiform neurofibromas and NF1-associated complications. Furthermore, this study will discuss the need for a specific MNF1 follow-up guideline.

## Methods

### The MNF1 cohort

Patients with MNF1 were included from the outpatient clinic at the Centre for Rare Diseases (CRD), Department of Paediatrics and Adolescent Medicine, Aarhus University Hospital, one of two Danish national specialist centers for NF1 covering the western part of Denmark. The center monitors patients with NF1 from infancy until old age, regardless of severity of NF1. There is free and equal access to healthcare for all residents in the Danish tax-based healthcare system.

### Protocol

We conducted a cross-sectional study. A medical chart review was performed. The inclusion criteria were a diagnosis of MNF1 by a pediatrician or clinical geneticist with expertise in NF1. The patient exclusion criteria were (1) positive *NF1* gene analysis without signs of mosaicism performed on DNA from blood, and (2) fulfillment of the NF1 clinical NIH diagnostic criteria [17]. A patient was defined as having unilateral MNF1 if he or she had one or more of the NIH diagnostic criteria limited to one area of the body, and the manifestations of NF1 did not cross the midline. A patient was defined as having bilateral MNF1 if she or he had one or more of the NIH diagnostic criteria in two or more discrete, non‐contiguous areas of the body [18]. A systematic data collection from the MNF1 cohort was completed. Data included demographic characteristics (age, sex, date of diagnosis), clinical features of NF1 according to the diagnostic criteria of NF1 [17]; pigmentary changes, neurofibromas, plexiform neurofibromas, Lisch nodules; optic pathway glioma, skeletal dysplasia and family history of NF1 and *NF1* analysis (if performed). We collected any additional information on associated NF1 complications such as skeletal abnormalities, development difficulties and malignancies.

Based on the clinical features, each patient was categorized according to the four MNF1 categories suggested by Ruggieri and Huson: (1) pigmentary changes only, (2) pigmentary changes and neurofibromas, (3) neurofibromas only, and (4) plexiform neurofibromas only [8].

Primary outcomes were the development of plexiform neurofibromas including the proportion of patients with MNF1 with plexiform neurofibroma and age at presentation. Secondary outcomes were the proportion of all clinical characteristics including NF1-associated complications and the results of *NF1* analysis (if performed).

### Data analysis

Data were collected and managed using the Research Electronic Data Capture (REDCap) tool hosted by Department of Clinical Medicine, Aarhus University. REDCap is a web-based platform designed to safe handling of data capture for research and automated export functions for data downloads [19, 20]. The clinical features including *NF1* analysis were studied retrospectively. Descriptive statistics were calculated using counts and proportions for categorical variables and medians and ranges for continuous variables.

### Literature on MNF1

A literature search was performed in PubMed in September 2020 using the following MESH terms: (((“Neurofibromatoses”[Mesh]) OR”Neurofibromatosis 1″[Mesh])) AND “Mosaicism”[Mesh])). We limited the search to articles written in English and published between 2013 and 2020. Since a systematic review on MNF1 between 1977 and 2012 was published in 2016, we chose to look at literature only published after 2013 [11]. The inclusion criteria were (1) case reports describing patients who had localized manifestations of NF1 according to MNF1 and (2) case series including adequate clinical information on each patient with MNF1. Adequate information was demographics; sex, age at presentation; and disease manifestations, type and location of manifestations, other conditions or complications associated with NF1, or/and results of *NF1* analysis (if performed).

## Results

### The MNF1 cohort

#### Clinical features

Seventeen patients, 11 females (65%), with MNF1 were identified. Eleven patients had unilateral MNF1. Six patients had bilateral MNF1; two had a negative *NF1* analysis on blood. The remaining four patients with bilateral MNF1 were all adults. All 17 patients had a negative family history of NF1. At inclusion, the median age was 37 years [4; 83]. Table 1 presents the MNF1 cohort according to the four MNF1 categories. Eight patients had only pigmentary changes, two patients had both pigmentary changes and neurofibromas, five patients had only neurofibromas and two patients had only plexiform neurofibromas. Patients with only pigmentary changes were younger at time of the MNF1 diagnosis with a median age of nine years [1; 16] compared to patients with only neurofibromas with a median age of 40 years [29; 77]. Patients with only plexiform neurofibromas had a median age of 28 years [25;31] at the date of MNF1 diagnosis; the plexiform neurofibromas were, however, generally identified before the MNF1 diagnosis when patients had a median age of 17 years [14;20].

**Table 1:** The MNF1 cohort from Centre for Rare Diseases (n = 17)

	Total	Pigmentary changes only	Pigmentary changes and neurofibromas	Neurofibromas only	Plexiform neurofibromas only
Patients, n	17	8	2	5	2
Females, n (%)	11 (64.7%)	6 (75%)	1 (50%)	3 (60%)	1 (50%)
Median age, y [range]	37 [4;83]	18 [4;37]	59 [51;66]	56 [37;83]	37 [33;40]
Median age at MNF1 diagnosis, y [range]	25 [1;77]	9 [1;16]	54 [46;61]	40 [29;77]	28 [25;31]
*CAL spots, in number of patients (%)*		8 (100%)	2 (100%)	0	0
Head and neck, n (%)		1 (12.5%)	–	–	–
Trunk, n (%)		7 (87.5%)	2 (100%)	–	–
Upper extremities, n (%)		2 (25%)	–	–	–
Lower extremities, n (%)		5 (62.5%)	1 (50%)	–	–
Pelvic area, n (%)		3 (37.5%)	–	–	–
Size of largest CAL spot (mm), median [range]		50 [13;150]	15	–	–
*Freckling, in number of patients (%)*		5 (62.5%)	0	0	0
Axillary unilateral, n (%)		4 (50%)	–	–	–
Inguinal unilateral, n (%)		1 (12.5%)	–	–	–
*Neurofibromas, in number of patients (%)*		–	1 (50%)	5 (100%)	0
Head and neck, n (%)		–	–	1 (20%)	–
Trunk, n (%)		–	1 (50%)	4 (80%)	–
Lower extremities, n (%)		–	–	2 (40%)	–
Pelvic area, n (%)		–	–	2 (40%)	–
Size of largest neurofibroma (mm), median [range]		–	4	40 [4;50]	–
*Plexiform neurofibromas, in number of patients (%)*		0	1 (50%)	2 (40%)	2 (100%)
Head and neck, n (%)		–	–	–	2 (100%)
Lower extremities, n (%)		–	1 (50%)	1 (20%)	–
Pelvic area, n (%)		–	–	2 (40%)	–
Median age by detecting of plexiform neurofibroma, y [range]		–	60	35 [30;40]	17 [14;20]
*Lisch nodules, in number of patients (%)*		1 (12.5%)	1 (50%)	0	0
Unilateral, n (%)		0	0	–	–
Bilateral, n (%)		1 (100%)	1 (50%)	–	–
*NF1 associated complications, in number of patients (%)*		4 (50%)*	1 (50%)**	4 (80%)***	0

*Scoliosis (n = 2), learning disability (n = 1), reading difficulties (n = 1), infantile autism (n = 1), ADHD (n = 1), delayed for age (n = 1), gross and fine motor function delays (n = 1), language delay (n = 1)

**Scoliosis and language delays (n = 1)

***Hypertension (n = 4), learning disability (n = 1)

NF1-associated complications were hypertension, scoliosis, learning disability, delay of language and cognition, infantile autism, attention deficit hyperactivity disorder (ADHD) and delay in gross and fine motor function development. In total, nine (53%) patients had one or more complications associated to NF1. Table 1 presents the complications at individual patient level. No hypertension diagnosis was related to pheochromocytoma or renal artery stenosis. Age of hypertension diagnoses was 22, 36 and 42 years and unknown in one patient since the patient had the diagnosis at the first visit at our centre at age 77. No patients were diagnosed with optic pathway glioma, bone dysplasia or malignant disease including MPNST at the time of data collection.

#### *NF1* variants

Six patients had had an *NF1* analysis performed. A variant was detected in two patients (Table 2). In patient no. 12, a disease-associated variant (c.3721C > T (p.Arg1241)) was detected in DNA from a neurofibroma, whereas the result of the analysis on DNA from blood was normal. This patient had two CAL spots, more than six neurofibromas and bilateral Lisch nodules. In patient no. 17, a pathogenic variant (c.5814_5815delTT) was detected in a mosaic form both in the DNA from a neurofibroma and in the DNA from blood. The only clinical feature of NF1 was more than six neurofibromas. Gonadal mosaicism was confirmed as the patient had a child with the same *NF1* variant in the germline (i.e. non-mosaic variant in DNA from blood) and generalized NF1. Two of the four patients with pigmentary changes only had genetic analyses performed. In one, a *SPRED1* negative result was reported and in one, information on *SPRED1* analysis was missing. The third patient, a minor, awaits analysis until older of age, and the fourth patient is not currently interested in genetic analysis.

**Table 2:** Results of the *NF1* analyses in the MNF1 cohort from Centre for Rare Diseases

Patient/type	Sample	Analysis method	Result of *NF1* analysis
10, Unilateral	Blood (B-lymphocytes)	MLPA analysis	Negative
12, Unilateral	Neurofibroma	NGS and MLPA	Disease-associated variant (c.3721C > T(p.Arg1241))
	Blood	–*	Negative
14, Unilateral	Blood and plexiform neurofibroma	NGS and MLPA	Negative incl. SPRED negative
15, Bilateral	Blood and CAL spot	–*	Negative
16, Bilateral	Blood	NGS and MLPA	Negative incl. SPRED negative
17, Unilateral	Blood and neurofibroma**	DNA sequencing (exon 31)	Pathogenic variant (c.5814_5815delTT)

MLPA = Multiplex Ligand-dependent Probe Amplification, NGS = Next Generation Sequencing

*Information on the analysis method was not available

**The level of mosaicism was estimated to be higher in DNA from the neurofibroma biopsy than in the DNA from blood, but without information on percentages

#### Review of the literature

**Fig. 1:**
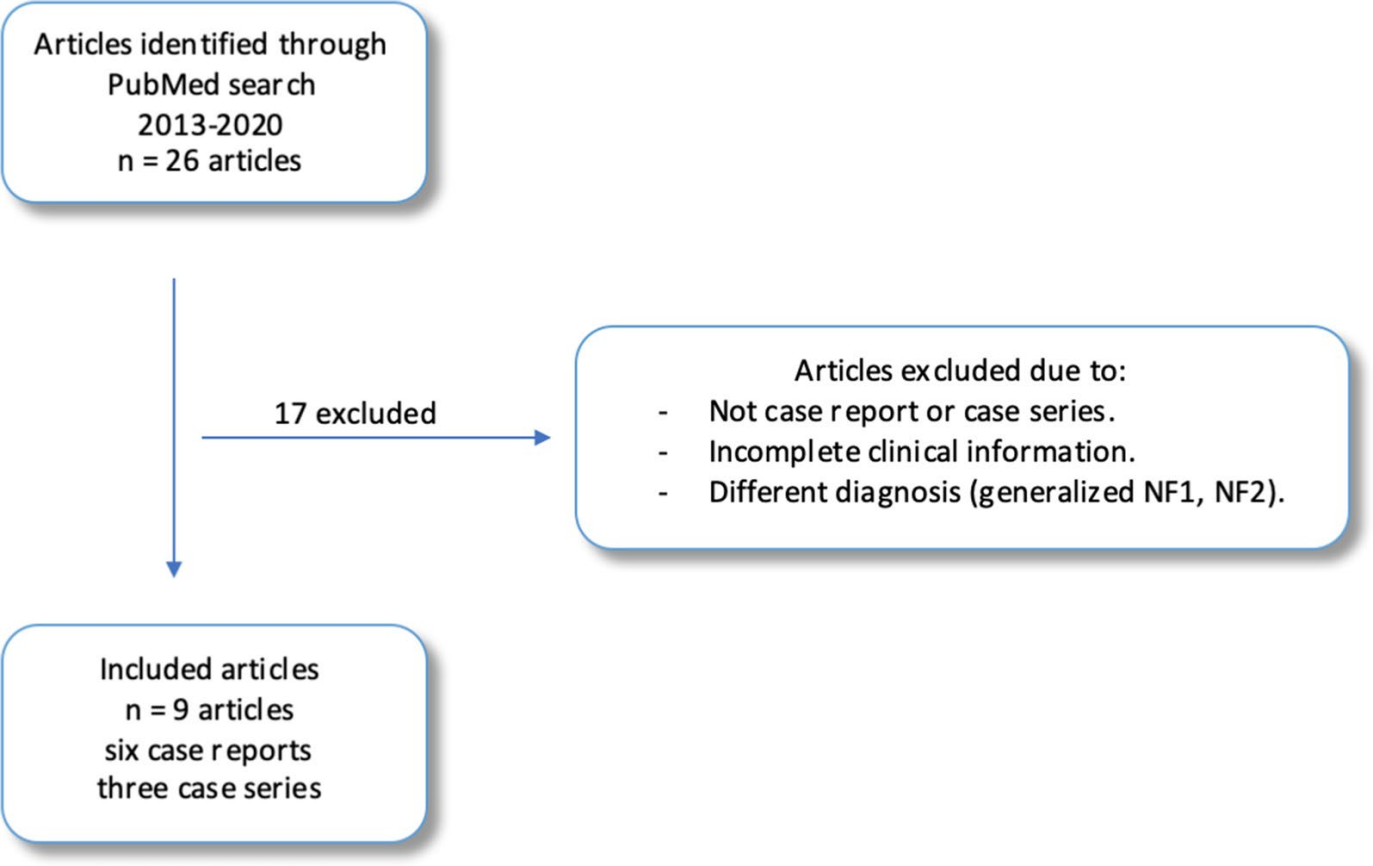
Study flow diagram

We identified 26 articles in our literature search in the PubMed database (Fig. 1). Nine articles were included in the study consisting of three case series and six case reports.

#### Clinical features

The patients with MNF1 included in the case series were recruited from hospitals or clinical centers and the patient cohorts were described in general [10, 21, 22]. In total, 126 cases were found in the three case series. The cases are presented in Table 3. Nineteen out of 126 (15%) cases had plexiform neurofibromas and in total, 23 NF1-associated complications were reported. The two most reported complications were skeletal abnormalities and learning disabilities followed by ADHD, language delay and intracranial vasculopathy.

**Table 3.** 126 Cases from three case series presented in total and individual articles

Total [10, 21, 22]	
Number of patients	n = 126
Mean age, years	Mean age = 16.44 ± 0.6 years
Females, n (%)	76 (60%)
Four categories	Pigmentary changes only (n = 79), neurofibromas only (n = 14), pigmentary changes and neurofibromas (n = 15), plexiform neurofibromas only (n = 18)
NF1-associated complications	language delay (n = 1), learning disability (n = 7), ADHD (n = 3), skeletal abnormalities [11] and intracranial vasculopathy (n = 1)
Lara-Corrales et al. [21]	
Number of patients	n = 60
Recruited from	Hospital for Sick Children, Toronto, Canada, in 1992–2012
Mean age, years	Mean age = 10.6 ± 4.6 years
Females, n (%)	32 (53%)
Four categories	Pigmentary changes only (n = 39), neurofibromas only (n = 9), pigmentary changes and neurofibromas (n = 2), plexiform neurofibromas only (n = 10)
NF1-associated complications	Learning disability (n = 7), bony changes (n = 6), seizures (n = 1)
Tanito et al. [10]	
Number of patients	n = 58
Recruited from	The Jikei University Hospital, in 2004–2007, and at the Jikei University Daisan Hospital, in 2007–2011
Mean age, years	Mean age = 23.4 [1;69] years
Females, n (%)	42 (72%)
Four categories	Pigmentary changes only (n = 32), neurofibromas only (n = 5), pigmentary changes and neurofibromas (n = 13), plexiform neurofibromas only (n = 8)
NF1-associated complications	Bone deformity (n = 3), language delay (n = 1)
Marwaha et al. [22]	
Number of patients	n = 8
Recruited from	Hospital for Sick Children, Toronto, Canada. Years unknown
Mean age, years	Mean age = 9.8 [1;16] years
Females, n (%)	2 (25%)
Four categories	Pigmentary changes only (n = 7), neurofibromas only (n = 0), pigmentary changes and neurofibromas (n = 1), plexiform neurofibromas only (n = 0)
NF1-associated complications	Sphenoid wing dysplasia (n = 1), intracranial vasculopathy (n = 1), tibial rotation (n = 1), ADHD (n = 3)

In total, seven cases with MNF1 were presented in the six case reports (Table 4). Patients presented in the following four groups; no patients with only pigmentary changes, three patients (43%) had neurofibromas only, three patients (43%) had pigmentary changes and neurofibromas, and one patient (14%) had plexiform neurofibromas only. No patients had NF1-associated complications, but one case had developed three malignancies not known to be directly related to NF1; renal cell carcinoma, mixed thyroid carcinoma and lentigo maligna [23]. Another case had a daughter with generalized NF1 and two unaffected children consistent with gonadal mosaicism [24].

**Table 4:** Seven cases from six case reports

Characteristics	All patients (n = 7)	Pigmentary changes only (n = 0)	Neurofibromas only (n = 3)	Pigmentary changes and neurofibromas (n = 3)	Plexiform neurofibromas only (n = 1)
Median age, years (range)	55 [35;72]	–	61 [40;72]	46 [35;66]	55
Female, n (%)	5 (71%)	–	2 (67%)	2 (67%)	1 (100%)
CAL spots, n (%)	2 (29%)	–	–	2 (67%)	–
Freckling, n (%)	2 (29%)	–	–	2 (67%)	–
Neurofibromas, n (%)	6 (86%)	–	3 (100%)	3 (100%)	–
Plexiform neurofibromas, n (%)	1 (14%)	–	–	–	1 (100%)
NF1 associated complications, n (%)	0	–	0	0	0

#### *NF1* variants

In the case series, 22 patients had undergone an *NF1* analysis performed on DNA from blood, CAL spots and plexiform neurofibromas [21, 22]. In 11 cases a pathogenic *NF1* variant was detected; an intragenic variant in nine cases and a microdeletion in two cases [21, 22]. Within the case reports, one patient had undergone *NF1* analysis and an atypical large *NF1* deletion was found in DNA from three plexiform neurofibromas [25].

## Discussion

In the absence of international guidelines on the follow-up on MNF1 and sparse publications on larger MNF1 cohorts, this study sought to investigate the severity of MNF1 with focus on plexiform neurofibromas in a cohort of children and adults and to perform an update of a literature review. This study is the first to present a Danish cohort of patients with MNF1 in relation to the current literature on MNF1. We found that the proportion of patients with MNF1 and plexiform neurofibromas within our cohort is higher (29%) than described in the literature reviewed in this article (15%) and that the diagnosis of a plexiform neurofibroma is often made before the actual MNF1 diagnosis. We also found that the phenotype severity of MNF1 on an individual level correlated to expected NF1 complications at the relevant age in some cases is similar to the phenotype seen in generalized NF1 with many NF1-associated complications.

We found a higher frequency of plexiform neurofibromas in our cohort (29%) than previously reported in MNF1 literature; 6.5% in children and adults and 4% in children, and 11.5% in a systematic review of 157 children and adults [8, 11, 18]. Especially the larger plexiform neurofibromas can cause moderate to severe morbidities such as pain and/or disfigurement. Interestingly, we found the median age at the diagnosis of plexiform neurofibromas was lower than the age at the actual MNF1 diagnosis, even though plexiform neurofibromas are believed to be congenital and most often detected in childhood [10]. This underlines that it can be challenging to establish the MNF1 diagnosis; moreover, the risk of developing plexiform neurofibroma after reaching adulthood cannot be neglected. It is important that physicians are aware that plexiform neurofibroma may be a sole manifestation of MNF1 and not just an idiopathic plexiform neurofibroma. Interestingly, the plexiform neurofibroma in patients with only plexiform neurofibromas was located at the head and neck of patients in our cohort. Ruggieri and Huson also found that plexiform neurofibromas were most frequently located at the head and neck (88%) in patients with only plexiform neurofibromas [8]. Lara-Corrales et al. found that 73% of all plexiform neurofibromas were located at the head and neck [21]. In our cohort, one patient had two plexiform neurofibromas. Lara-Corrales et al. found that 17% of patients who previously had plexiform neurofibromas developed new plexiform neurofibromas after a mean follow-up of 3.7 ± 3.3 years [21]. A long-term follow-up including both children and adults with MNF1 could be beneficial to increase the clinical understanding of the development of plexiform neurofibromas. So far, only children with generalized NF1 and congenital plexiform neurofibromas qualify for treatment with selumetinib. Even if a diagnosis of MNF1 could qualify for treatment, patients with MNF1 are often diagnosed with both MNF1 and plexiform neurofibromas after reaching adulthood. In the light of the rather high number of plexiform neurofibromas in the MNF1 population, it could be advocated that patients with MNF1 have the same need of assessment for treatment with selumetinib.

Within our cohort, nine out of 17 patients (53%) had NF1-associated complications. Six of the nine these had cognitive impairments. Cognitive impairment has previously been shown to be less frequent in patients with MNF1 [8, 10, 18], but within both our cohort and the literature review we found cognitive impairment to be just as frequent as somatic complications. Any degree of cognitive impairment can be challenging to an individual, and the challenges found in this study are all biopsychosocial factors influencing quality of life. Furthermore, several studies have reported reduced quality of life in children, adolescents and adults in the NF1 population [6].

Assessing the age and phenotype of the MNF1 diagnosis, our data show that the median age at diagnosis of patients with pigmentary changes only (n = 8) was nine years, whereas the median age at diagnosis of patients with plexiform neurofibromas (n = 2) and neurofibromas only (n = 5) was 28 years and 40 years, respectively. According to the literature on MNF1, pigmentary changes develop in early childhood, followed by the appearance of plexiform neurofibromas in later childhood; cutaneous neurofibromas develop in adulthood [8]. Since the patients in our cohort with pigmentary changes only had a median age of 18 years at inclusion, they have a risk of developing neurofibromas later in life. The presence of a mild phenotype with only few neurofibromas may not lead to seeking medical attention; this may suggest that neurofibromas are underreported in the cohorts and that MNF1 is underdiagnosed in the general population. In support of this, Listernick et al. proposed the misdiagnosis of MNF1 as generalized NF1 in cases of significant neurofibromas [18].

Only six patients in our MNF1 cohort had had an *NF1* analysis performed and a variant was only detected in two patients; no patients presented with *NF1* microdeletions. In our review of the literature, 11 variants in 22 cases were detected of which two were microdeletions. Kehrer-Sawatzki et al. did not find any patients with MNF1 with *NF1* microdeletions to have more severe phenotypes [26]. Any other genotype–phenotype correlation in MNF1 has not been shown [22, 27]. Individuals with MNF1 are at risk of gonadal mosaicism, which gives a risk of offspring with generalized NF1 and any NF1-associated complication. Within our cohort, one patient had a child with generalized NF1 (0.6%) and in the previously published literature, frequencies were 2.5% and 6.4% [8, 11]. A sperm donor with no diagnosis of NF1 or MNF1 fathered nine children out of 23 (39%) with NF1 and had an estimated 20% gonadal mosaicism for NF1 [28]. This shows that an estimate of gonadal mosaicism in semen is not equal to the true proportional risk of offspring with NF1.

This study has some limitations. Our cohort consisted of a small group of patients, which makes it difficult to draw conclusions about the general MNF1 patient group. In addition, statistical calculations were not possible because of heterogenic data between our cohort and the reviewed literature and within the literature itself. *NF1* analyses were only performed in six patients in our cohort; hence, it was not possible to categorize the cohort according to the genotype. When comparing the frequencies of complications between MNF1 studies one need to note whether the frequency was reported in a follow-up study or based on cases in a cross-sectional study. Most of the literature on MNF1 are on cases; hence, the distribution of ages within the cases and cohorts needs to be taking into account, since both NF1 and MNF1 manifestations evolve over age.

The diagnostic process of MNF1 was a challenge as this process may vary among physicians and across countries indicating the need for international clinical diagnostic criteria for MNF1. Listernick et al. used the criteria used in our study as well [18]. However, even with these criteria on bilateral MNF1 for patients with one or more of the NIH diagnostic criteria in two or more discrete, non‐contiguous areas of the body, it can still be a challenge clinically to rule out generalized NF1. Lara-Corrales et al. suggested that the ideal way to confirm the diagnosis of MNF1 is a positive *NF1* analysis on DNA from affected tissue together with a negative *NF1* analysis on DNA from blood [21]. However, one of the patients from our MNF1 cohort had a positive *NF1* analysis on DNA from both affected tissue and blood, though in a mosaic state and two patients had a negative *NF1* analysis on DNA from a plexiform neurofibroma and from a CAL spot, respectively. Tanito et al. proposed that Lisch nodules should be unilateral or ipsilateral to the affected area on the skin to support the diagnosis of MNF1 [10]. Contradictory, both patients with Lisch nodules from our MNF1 cohort presented with bilateral Lisch nodules. Currently, a group of European and North American NF1 experts are revising the NF1 diagnostic criteria including the criteria of MNF1 [29].

## Conclusion

Our study on a MNF1 cohort and a review of literature showed that NF1-associated complications such as plexiform neurofibromas and cognitive impairment are present relatively often in patients with MNF1, and that debut of complications can occur rather late in life. Our study is supportive of recent literature suggesting that patients with MNF1 should be followed by the same clinical guidelines as patients with generalized NF1 in both childhood and adulthood. Moreover, there should be a greater emphasis among physicians on cognitive challenges in patients with MNF1. An *NF1* analysis is advisable both as a diagnostic tool and as part of genetic counseling as patients with MNF1 could have gonadal involvement. To develop a specific MNF1 follow-up guideline, there is a need for an international consensus on the diagnostic criteria for MNF1 and a follow-up study conducted in a larger MNF1 cohort.

## Data Availability

Could be available from the corresponding author on reasonable request.

## Abbreviations

ADHD: Attention deficit hyperactivity disorder
CAL: Café-au-lait
CRD: Centre for Rare Diseases
MLPA: Multiplex Ligand-dependent Probe Amplification
MNF1: Mosaic neurofibromatosis type 1
NF1: Neurofibromatosis type 1
NGS: Next Generation Sequencing
REDCap: Research Electronic Data Capture
MPNST: Malignant peripheral nerve sheet tumor
